# Long-term remission of prostate cancer with extensive bone metastases upon immuno- and virotherapy: A case report

**DOI:** 10.3892/ol.2014.2588

**Published:** 2014-10-06

**Authors:** VOLKER SCHIRRMACHER, AKOS-SIGMUND BIHARI, WILFRIED STÜCKER, TOBIAS SPRENGER

**Affiliations:** 1 Immunological and Oncological Center, Cologne, Germany; 2 German Cancer Research Center, Division of Translational Immunology, Heidelberg, Germany

**Keywords:** prostate cancer, Newcastle disease virus, hyperthermia, dendritic cell vaccination, immunotherapy

## Abstract

The present study reports the case of a patient with hormone-refractory metastatic prostate cancer who had failed standard therapy, but then achieved complete remission following combined treatment with local hyperthermia (LHT), Newcastle disease virus and dendritic cell (DC) vaccination, which was an unusual combination. In August 2005, the patient underwent a radical prostatectomy. Despite standard treatment, the patient developed progressive bone metastases and stopped conventional therapy in June 2007. Starting in October 2007, the patient was treated with LHT, oncolytic virotherapy and DC vaccination. Prostate-specific antigen (PSA)-levels, with the highest level of 233.8 ng/ml in January 2008, decreased to 0.8 ng/ml in late February 2008. In March 2008, a reduction in bone metastases could be detected by positron emission tomography/computed tomography. Since then, the PSA levels have remained low and the patient is doing well. The treatment induced a long-lasting antitumor memory T-cell response. This possibly explains the long-term effectiveness of this novel experimental combined treatment approach.

## Introduction

Prostate cancer is the most common malignant tumor in males (1). Conventional treatment, including surgery and radiotherapy, has potential secondary effects, such as impotence or incontinence, that can greatly impair quality of life. By contrast, specific immunotherapy has no severe side-effects, as it affects only the malignant cells and spares the healthy tissue. Dendritic cell (DC) vaccination is an important immunotherapeutic strategy. Oncolytic virotherapy and hyperthermia can have synergistic functions with immunotherapy (2).

The approval of the first therapeutic anticancer vaccine, sipuleucel-T, by the Food and Drug Administration for the treatment of metastatic hormone-refractory prostate cancer in April 2010 has enforced a new era of immunotherapy (3). In a phase III trial, following vaccination with activated autologous DC, a prolonged overall survival time was demonstrated in patients suffering from castration-resistant prostate cancer (4). In addition, a number of other clinical trials have reported the clinical benefit of DC vaccination (5).

Another promising approach is the use of oncolytic viruses that preferentially infect tumor cells. Newcastle disease virus (NDV) is an avian RNA paramyxovirus with a high safety profile in cancer patients. The three properties that make NDV suited for fighting human cancer are its tumor-selective replication, antitumor cytotoxicity and immunostimulation (6).

Hyperthermia has been used for the treatment of a diverse range of solid tumors. Various techniques for the application of heat have been developed. The cellular effects that have been described include the induction of apoptosis and the expression of heat shock proteins (HSPs) (7). Also, synergistic effects of hyperthermia in combination with chemotherapy and irradiation have been observed (8). The present study reports the effects of combining hyperthermia with oncolytic virotherapy and DC-based immunotherapy.

## Case report

### Case history

**Figure 1:**
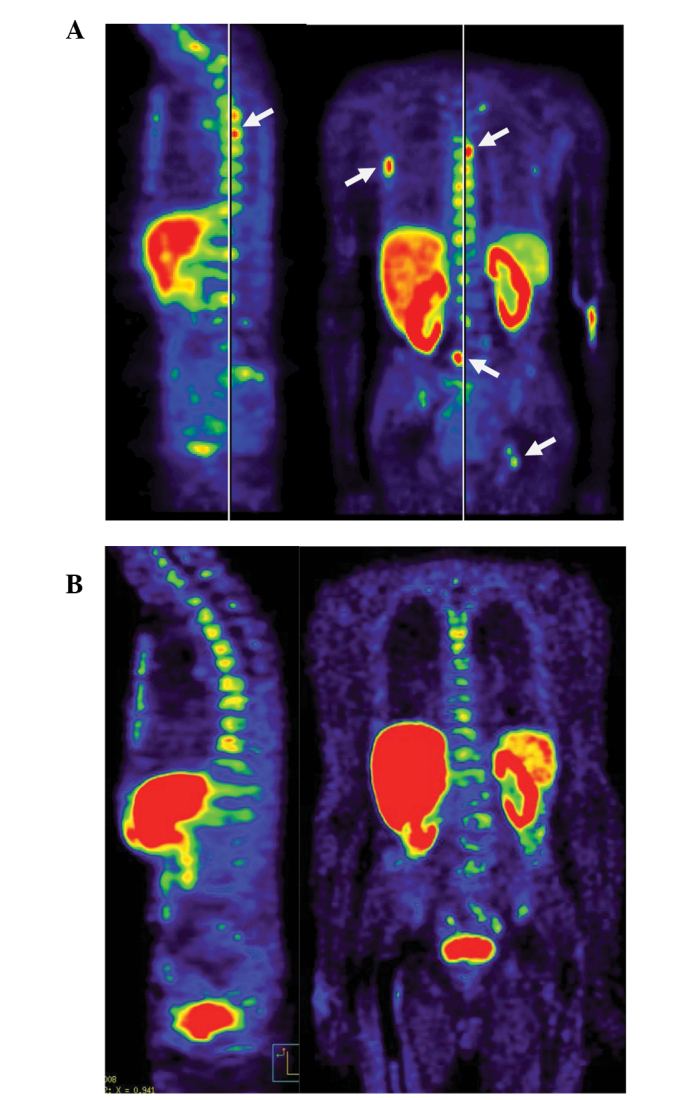
^18^F-choline-positron-emission-tomography/computed tomography scans. (A) From September 2007, prior to immunotherapy. Multiple focal tracer enhancements in the spine, 5th right rib and pelvis. (B) From March 2008, following immunotherapy. Distinct regression of skeletal metastases, no more focal enhancement observable. Mild diffuse tracer enhancement in thoracic spine, representing low remaining metabolic activity.

**Figure 2:**
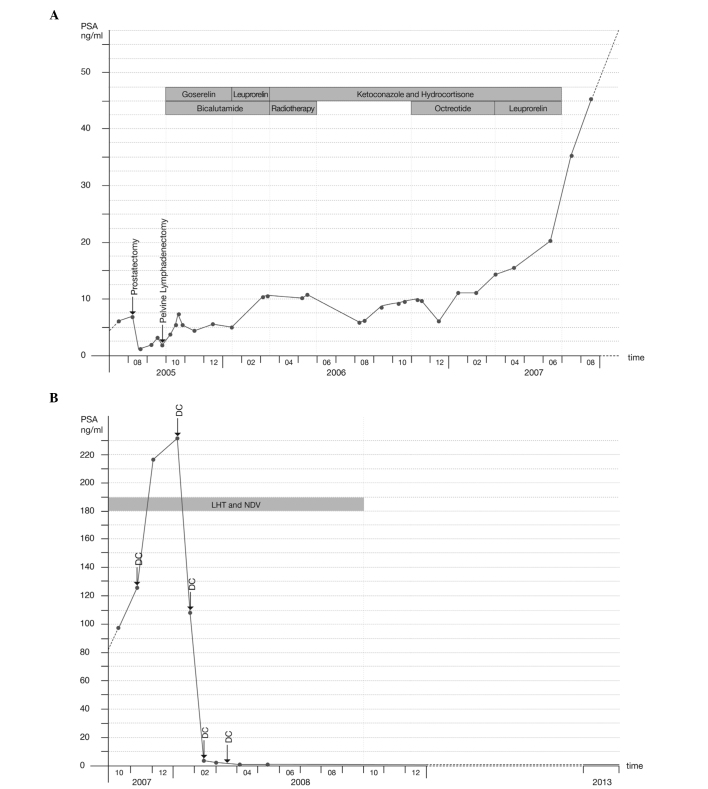
Prostate-specific antigen (PSA) levels from the blood (A) under conventional therapy and (B) under immunotherapy. DC, dendritic cell vaccination; LHT, local hyperthermia; NDV, Newcastle disease virus.

In October 2007, a 75-year-old patient presented at the Immunological and Oncological Center (Cologne, Germany) with progressive, hormone-refractory prostate cancer, with a prostate-specific antigen (PSA) doubling time of 65 days and the presence of bone metastases. The patient had previously undergone a radical prostatectomy in August 2005, at the time of the initial diagnosis. The post-surgical staging was pT3b pNX L1 V1 R0, with a Gleason Score of 9 (5+4). In September 2005, a bilateral pelvic lymphadenectomy was performed, which demonstrated no evidence of metastases (0/32). In October 2005, the patient started androgen suppression with goserelin (3.6 mg, once a month) and bicalutamide (50 mg, once a day), and in January 2006, the goserelin was switched to leuprorelin (10.72 mg, once every three months). Despite treatment, the patient’s PSA levels rose and a subcranial bone metastasis developed in March 2006. The tumor was classified as hormone-refractory and the androgen suppression was discontinued. Between March and May 2006, the patient underwent palliative radiotherapy with 45 Gy (30×1, 5 Gy), leading to a decrease in PSA levels from 11.6 to 6.5 ng/ml (following prostatectomy PSA is normally undetectable). Between March 2006 and June 2007, the patient was treated with ketoconazole (3×400 mg, once a day) and hydrocortisone (morning dose, 20 mg and evening dose, 10 mg, daily) in an attempt to block adrenal and testicular androgen synthesis. Within the scope of a clinical trial, octreotide was administered experimentally between November 2006 and March 2007. Due to rising PSA levels (from 40.8 ng/ml to 60.5 ng/ml), octreotide was discontinued in favor of another attempt with leuprorelin (10.72 mg, once every three months) between March and June 2007. Upon termination of androgen deprivation, rising testosterone levels (from 0.18 ng/ml in October 2007 to 6.25 ng/ml in May 2013; normal range, 2.14–8.27 ng/ml) were documented. In April 2007, a scintigram revealed bone metastases in two ribs and the left sacrum. From July 2007, the PSA levels rose further, most likely due to the progress of osseous metastases. In September 2007, positron emission tomography/computed tomography (PET/CT) revealed extensive, disseminated bone metastases of the entire spine, pelvis, right humerus and ribcage [ECAT EXACT 47, (Siemens Medical Systems, Erlangen, Germany); visualised using MPI-Tool, (Advanced Tomo Vision GmbH, Kerpen, Germany)] (Fig. 1A). The university hospital at which the patient was being treated advised the commencement of chemotherapy, but the patient decided to begin immunotherapy. By October 2007, the patient’s PSA level had risen to 98.1 ng/ml (Diamond Select GEMINI GXL 16 PET/CT System, Philips, Eindhoven, Netherlands; visualised using Syntegra Imaging software version 2.1, Philips)(Fig. 2A).

### Immunotherapy

Between October 2007 and June 2008, the patient was treated at the Immunological and Oncological Center (Cologne, Germany) with locoregional hyperthermia of the pelvis and thorax, and systemic oncolytic NDV virotherapy approximately twice a week. In addition, in November 2007, the patient received two sessions of local hyperthermia (LHT) of the occiput.

Between November 2007 and March 2008, the patient received five vaccinations with autologous antigen-pulsed DCs combined with moderate whole-body hyperthermia. Between July and September 2008, the patient was treated with LHT of the pelvis and thorax, and oncolytic NDV virotherapy once a month to sustain the immune response. After September 2008, no further immunological treatment was considered necessary.

Hyperthermia was administered with the Oncothermia EHY-2000 device (Oncotherm GmbH, Troisdorf, Germany) with a radiofrequency of 13 MHz. In total, the patient received 46 hyperthermia treatments to the thorax, 54 to the pelvis and two to the occiput. The duration of the sessions was 50 min each, starting at 60 W and increasing to 130 W. Intravenous administration of 109 plaque-forming units of NDV (strain MTH-68) were provided per session.

DCs were differentiated from autologous monocytes with granulocyte-macrophage colony-stimulating factor and interleukin-4. Immature DCs were pulsed with a lysate from NDV-infected DU145 prostate carcinoma cells, termed the oncolysate. Subsequent to maturation, the cells were administered intradermally simultaneously with interferon-γ (IFN-γ; 0.1 mg), followed by moderate whole-body hyperthermia (temperature, 38.5–39.0°C; infrared device, Heckel-HT2000; Heckel Medizintechnik GmbH, Esslingen, Germany).

### Outcome and follow-up

By early January 2008, the PSA levels had reached a maximum of 233.8 ng/ml. In late January and throughout February, the PSA levels decreased to 0.8 ng/ml. In March 2008, a reduction in the bone metastases was detected by PET/CT (Fig. 1B). The PSA level has remained low up to the present time and was <0.03 ng/ml in December 2013 (Fig. 2B).

Since there was sustained cancer remission, the patient was tested for the development of an immunological antitumor memory T-cell response. An Enzyme-Linked ImmunoSpot assay (Autoimmun Diagnostika GmbH, Strassberg, Germany) was performed in July 2011 to quantify the numbers of T cells that were secreting IFN-γ upon short-term (48-h) contact with oncolysate-pulsed autologous DCs. Overall, 150±10 patient blood-derived circulatory T cells per 100,000 T cells were found to respond, but without oncolysate pulsing there was only a background response of T cells and DCs of 2±1 cells.

Written informed consent was obtained from the patient for the publication of this case report and the accompanying images.

## Discussion

The first conclusion that can be made from this case study is that immunotherapy can have an impact on metastatic prostate cancer. The described procedures provoked no relevant side-effects. The successful experimental approach involved a combination of LHT, oncolytic virotherapy and DC vaccination. It is likely that the observed immunological memory T-cell response contributed to the long-term effects of treatment, as has been described recently for colon carcinoma (9).

The second conclusion that can be made is that this case is of importance and relevance, as it demonstrates that immunotherapy is not restricted to early-stage cancer, as has been previously assumed (10). The results were achieved by a novel and scientifically well-founded combination of biological (NDV and DC) and physical (LHT) treatment procedures that produce synergistic effects. The strategy is not restricted to a particular type of cancer and may thus have broad implications for clinical oncology in general.

Lately, the traditional therapy for prostate cancer, in particular radical prostatectomy, has been questioned (11). Generally, the presence of metastases should be determined prior to prostatectomy. The remaining and quickly rising post-surgical PSA-levels in the present patient suggested that there had been metastases at the time of the surgical intervention. Therefore, in this particular case, the prostatectomy had not only been in vain, but had also led to permanent incontinence. All further conventional therapies had failed. Apart from the immunotherapy described, the patient was not treated with any other conventional or alternative therapy and did not undergo any significant change in lifestyle.

In the following analysis, an explanation for the success of this novel combined treatment is outlined. To avoid possible immune escape mechanisms, including antigen shift and the induction of tolerance by the tumor, a DC vaccine with multiple prostate carcinoma antigens was used and two strategies for the introduction of danger signals into the tumor cells were utilized, consisting of oncolytic viruses and hyperthermia.

NDV infection introduces foreign viral RNA, activating the endosomal Toll-like receptor-3, cytoplasmic retinoic acid-inducible gene 1 and the plasma membrane-expressed viral hemagglutinin-neuraminidase proteins, leading to the induction of IFN-α and -β, and to the potentiation of T-cell-mediated antitumor immunity (10). The rationale for the combination of virus infection with hyperthermia was an expected synergy, as LHT has been reported to enhance virus tumor targeting and replication (2,12). In addition, viral infection and hyperthermia each cause an endoplasmic reticulum stress response (5,13), change the expression of HSP 70/90 and the surface properties (calreticulin) of tumor cells, and induce immunogenic tumor cell death mechanisms (14). This leads to antigen uptake by host DCs, cross-presentation of autologous tumor antigens and priming of specific T cells. Subsequent vaccination with oncolysate-pulsed DCs results in further priming and activation of T-cell antitumor immunity. Ideally, this elicits an effective antitumor cytotoxic reaction and leads to long-lasting antitumor T-cell memory. DC vaccination was combined with moderate whole-body hyperthermia, as DC function can be enhanced at elevated temperatures (15). More than three years after the DC vaccination, a good antitumor memory T-cell response was detected in the present patient.

Taken together, the combination of different immunotherapeutic strategies led to a surprising therapeutic success in the patient. In a different patient with inoperable metastasized prostate carcinoma, treated at the Immunological and Oncological Center, a similar strategy has led to sustained disease stabilization over a time-period of more than four years. The combination of DC vaccination, oncolytic NDV application and hyperthermia appears to be effective and deserves further investigation.

Single case studies, such as the present study, can provide important innovation and additions to our medical knowledge. Single case use, as approved by the German Pharmaceuticals Act (16), is important for patients and for medical progress.

## Abbreviations

DC: dendritic cell
HSP: heat shock protein
IFN: interferon
LHT: local hyperthermia
NDV: Newcastle disease virus
